# Craniofacial and Intracranial Langerhans Cell Histiocytosis

**DOI:** 10.5334/jbsr.2245

**Published:** 2020-10-23

**Authors:** Benjamin Leenknegt, Nele Herregods, Marc Lemmerling

**Affiliations:** 1 University Hospital Ghent, BE; 2 AZ Sint-Lucas Ghent, BE

**Keywords:** Langerhans Cell Histiocytosis, Osteolytic Bone Lesion, Child

## Abstract

**Main Teaching Point:** Multiple osteolytic calvarial lesions in a child raise suspicion of Langerhans cell histiocytosis.

## Case

**Figure 1:**
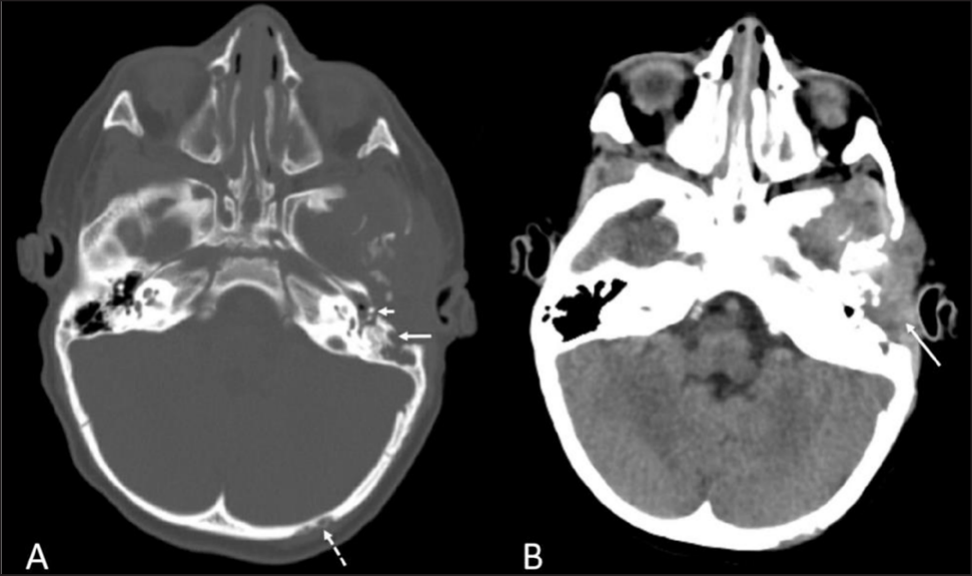


**Figure 2:**
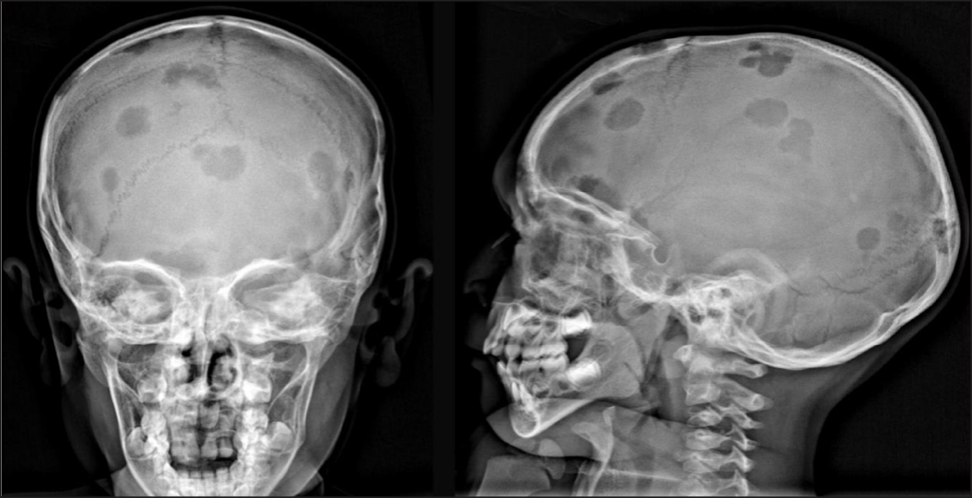


**Figure 3:**
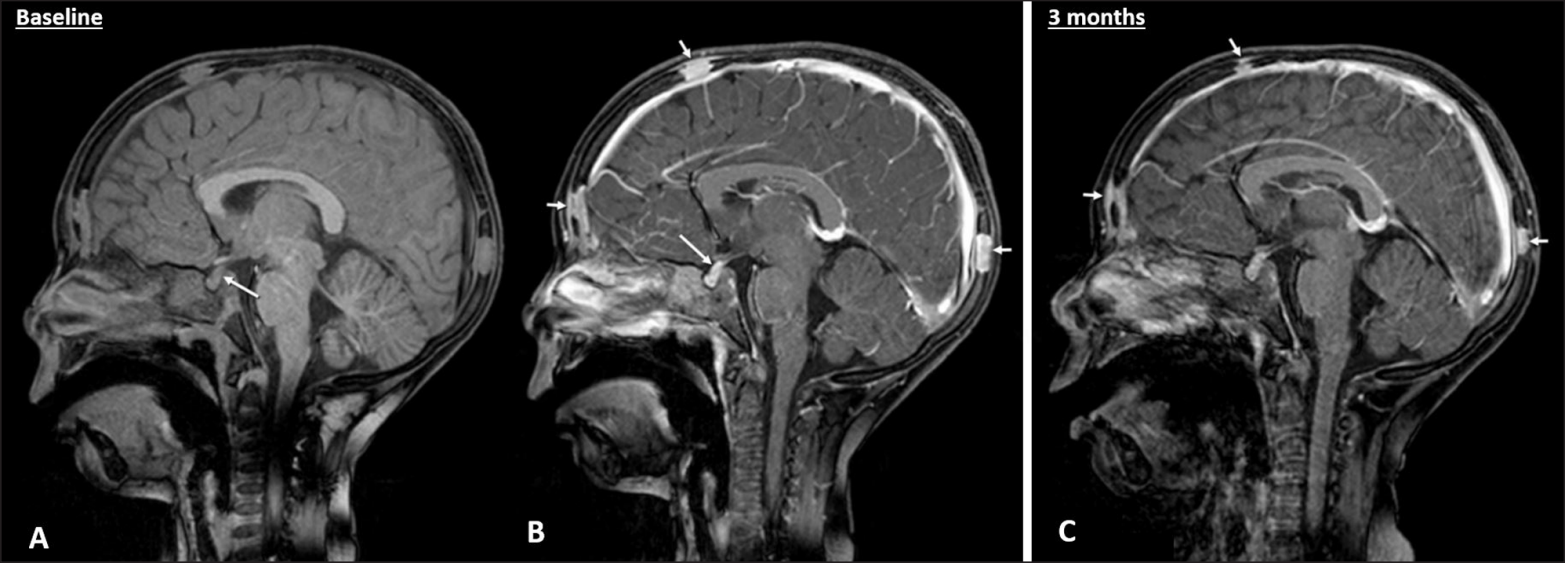


A four-year-old patient presented to the otorhinolaryngology department with left-sided otalgia and otorrhagia. On clinical examination, the left external auditory canal appeared edematous and hemorrhagic. Computed tomography (CT) of the temporal bone was performed, which demonstrated an osteolytic process in the left anterolateral mastoid (Figure 1A, long arrow), the middle ear (Figure 1A, short arrow) and in the left occipital bone (Figure 1A, dashed arrow). A soft tissue mass was present in the left auditory canal (Figure 1B). Additional CT of the brain demonstrated multiple similar bone lesions. Skeletal survey with X-ray confirmed the presence of the osteolytic lesions in the calvaria (Figure 2), but did not depict other lesions in the vertebra or the long bones. On brain magnetic resonance imaging (MRI), the normal high signal intensity of the posterior pituitary on T1-weighted images was absent (Figure 3A). After intravenous contrast, the bone lesions enhanced avidly (Figure 3B). The pituitary stalk appeared thickened (Figure 3B). Biopsy of a calvarial lesion confirmed the diagnosis of Langerhans cell histiocytosis and treatment with Vinblastine and Prednisolone was initiated. Follow-up imaging after three months (Figure 3C) showed a significant decrease in volume of the bony lesions.

Langerhans cell histiocytosis (LCH) is a rare hematologic disorder, characterized by proliferation of antigen-presenting dendritic cells called Langerhans cells. Incidence is estimated at 2–20 per million, with a slight male predilection and a peak between one and three years of age. Depending on the number of lesions and systems involved, the disease is composed of three categories. Eosinophilic granuloma is limited to one or a few bones with potential lung involvement. Hand-Schüller-Christian disease involves multiple bones, the reticuloendothelial system and the pituitary gland. Letterer-Siwe disease is disseminated disease with fulminant clinical course. Osseous lesions are the most common manifestation of LCH, most often in flat bones such as the skull, mandible, ribs, pelvis, and spine. On imaging, bone lesions appear as osteolytic defects (“punched-out lesions”). On radiographs, calvarial lesions often demonstrate a double contour caused by asymmetrical involvement of the inner and outer tables, called the “hole within a hole” or “bevelled edge”-sign. MRI is superior in the evaluation of an associated soft-tissue mass or dural invasion. On MRI, osseous lesions most often demonstrate intermediate T1 and increased T2 signal intensity and avid contrast enhancement, as was the case in our patient. Involvement of the hypothalamic-pituitary axis is characterized by an absence of high signal intensity of the posterior pituitary on T1-weigthed imaging and a thickening and enhancement of the pituitary stalk. Both findings were also present in our case. In the absence of organ dysfunction, prognosis of children with either localized or multifocal LCH is excellent [1].
